# Highly transparent triboelectric nanogenerator for harvesting water-related energy reinforced by antireflection coating

**DOI:** 10.1038/srep09080

**Published:** 2015-03-13

**Authors:** Qijie Liang, Xiaoqin Yan, Yousong Gu, Kui Zhang, Mengyuan Liang, Shengnan Lu, Xin Zheng, Yue Zhang

**Affiliations:** 1 State Key Laboratory for Advanced Metals and Materials, School of Materials Science and Engineering, University of science and technology Beijing, Beijing. 100083, China; 2 Key Laboratory of New Energy Materials and Technologies, University of Science and Technology Beijing, Beijing. 100083, China

## Abstract

Water-related energy is an inexhaustible and renewable energy resource in our environment, which has huge amount of energy and is not largely dictated by daytime and sunlight. The transparent characteristic plays a key role in practical applications for some devices designed for harvesting water-related energy. In this paper, a highly transparent triboelectric nanogenerator (T-TENG) was designed to harvest the electrostatic energy from flowing water. The instantaneous output power density of the T-TENG is 11.56 mW/m^2^. Moreover, with the PTFE film acting as an antireflection coating, the maximum transmittance of the fabricated T-TENG is 87.4%, which is larger than that of individual glass substrate. The T-TENG can be integrated with silicon-based solar cell, building glass and car glass, which demonstrates its potential applications for harvesting waste water energy in our living environment and on smart home system and smart car system.

Harvesting energy from green and renewable energy resources, such as solar, heat, and mechanical vibration has attracted increasing interest in the past decade not only for meeting the growing energy consumptions, but also for realizing the self-powered sensor systems^1^,^2^,^3^,^4^,^5^,^6^. Approaches based on various physical mechanisms, such as piezoelectric^7^,^8^,^9^,^10^, electrostatic^11^,^12^,^13^, and electromagnetic effects^14^,^15^, have been used to scavenge energy in our environment. The triboelectric effect^16^,^17^ is one of the most universal phenomena in our daily life, and it can explain most daily static electricity that generated from mechanical contact. It has been regarded as an undesirable effect for electronic systems due to the potential hazards. Though the mechanism of triboelectric effect is still in debate^18^,^19^,^20^, recently, triboelectric nanogenerator (TENG)^21^,^22^,^23^,^24^,^25^,^26^,^27^ based on triboelectric effect have been demonstrated to be a cost-effective, reliable, and extremely efficient device to convert mechanical energy to electricity. Energy conversion is achieved with the coupling of triboelectric effect and electrostatic induction: periodic contact and separation between materials that differed in polarity of triboelectricity yield a potential drop which will drive electrons to flow through an external load and produce continuous outputs.

Three fundamental operation principles of the TENG have been developed and show their potential applications, containing vertical contact-separation mode^28^, single-electrode mode^29^, and in-plane sliding mode^30^,^31^. For traditional TENG, two solid materials are essential and dry condition is necessary to keep its high output. Nevertheless, the water-related energy in the environment, such as waterfalls, rainwater and ocean waves have huge amounts of energy, which is inexhaustible, renewable and not limited by daytime, weather and climate. The flowing water carry two types of energy: one is the mechanical energy from the motion of the flowing water; the other is electrostatic energy that is produced from the tribo-charges during the contact electrification process with air or other materials. Previous studies^32^,^33^,^34^,^35^ have shown that the triboelectric nanogenerator can be used to harvest water-related energy.

Polytetrafluorethylene (PTFE) have been largely considered for applications of high-performance electronic and electret due to the combination of technologically attractive properties including low friction coefficient, high chemical and thermal stability, low dielectric constant, good mechanical strength and excellent plasticity. Moreover, PTFE is positioned extremely negative in the triboelectric series^36^ and is widely used in triboelectric nanogenerator.

The transparent characteristic is an important component in the new optoelectronic and electronic devices, and plays a key role in practical applications in our daily life. Some studies^37^,^38^,^39^ on transparent nanogenerators have been reported, and almost all of them are used to harvest mechanical energy. Here in this work, we designed a T-TENG to collect the electrostatic energy of water. The T-TENG is operated under single-electrode mode, which shows its convenience for scavenging energy from a continuously moving or flowing object. The thinnest film with thickness of 1 μm is prepared and the overlap of the hydrophobic PTFE film with the thickness less than 3 μm makes the transmittance of the fabricated device larger than that of the glass substrate, instead of smaller than that. With the acting as an antireflection coating of the PTFE film, the maximum transmittance of the fabricated device with PTFE film thickness of 1 μm is 87.4%, while the transmittance of the glass substrate is 83.4%. With the flow rate of the tap water of 93 ml/s, the output peak-to-peak open-circuit voltage and current density of the T-TENG can reach 10 V and 2 μA/cm^2^, respectively. The instantaneous output power density of the T-TENG is 11.56 mW/m^2^ when connected to a load resistor of 0.5 MΩ. The rectified outputs were applied to charge the commercial capacitors and drive light emitting diode. Our study shows the great potential of utilizing the T-TENG for harvesting the energy from flowing water and pushes forward an important step toward the practical applications of the TENG.

## Results

### Device structure

**Figure 1:**
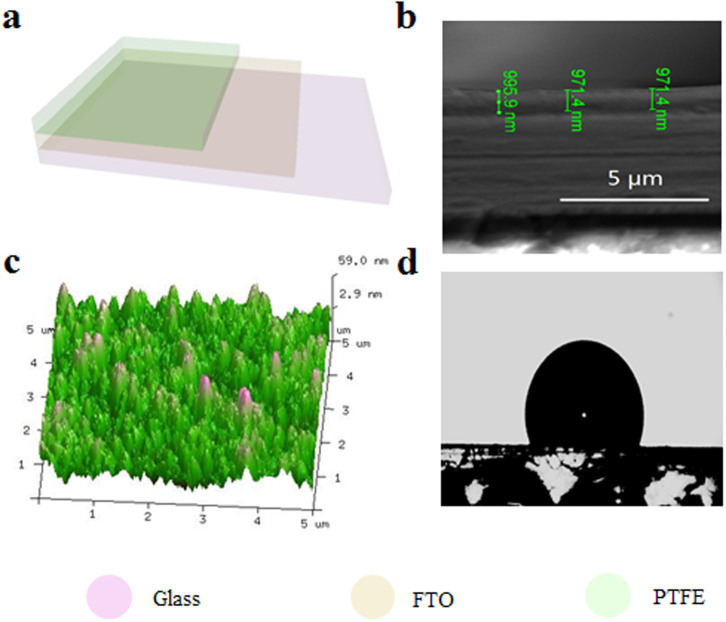
Structure illustration of the T-TENG. (a) Schematic diagram of the fabricated T-TENG. (b) Scanning electron microscopy (SEM) image of cross section of the T-TENG. (c) Atomic Force Microscope (AFM) image of the surface topography of the as-prepared PTFE film. (d) Contact angle of the PTFE film.

A schematic diagram of the fabricated T-TENG is shown in Figure 1a. The T-TENG is structurally composed of a rectangular glass, fluorine-doped tin oxide (FTO) electrode and a PTFE film, where glass is selected for the substrates due to its decent strength, high transparency and good machinability. Figure 1b illustrates the cross-sectional scanning electron microscopy (SEM) image of the fabricated T-TENG, where the typical PTFE film with thickness of 1 μm is presented (Figure 1b). The surface of the PTFE was characterized by the atomic force microscope (AFM), showing that the surface morphology of the PTFE thin film is composed of irregular nanostructures with roughness of tens of nanometers, as illustrated in Figure 1c. The PTFE thin films with thicknesses of 10 μm, 3 μm, 2 μm, 1.5 μm, 1 μm were prepared by spin-coating PTFE suspension with contents of 60%, 30%, 20%, 15%, 10%, respectively. The size of the effective working area of the T-TENG is 2.5 cm * 2 cm. The fabrication process is straightforward without sophisticated equipments and processes, demonstrating that this approach can be utilized to prepare large-scale T-TENG which is very important for the T-TENG to achieve practical applications in our environment. The detailed fabrication process will be presented in the Method Section.

In this paper, PTFE is chosen not only because the set of attractive properties mentioned above, but also because PTFE is a fluoropolymer with very low surface energy. Hydrophobic surfaces possess great advantages in self-cleaning, antisticking, de-icing and anticontamination. Besides, the hydrophobic ability of the film is a critical factor for the performance of the TENG. The hydrophobicity of the as-prepared PTFE film was investigated by measuring the contact angle of water drops. The measured typical contact angle of PTFE film is around 117°, as shown in Figure 1d. Compared to the smooth PTFE film with a contact angle^40^ of 108°, the slight increase of contact angle is caused by the rough surface of the film comprised of nanostructures (as shown in Figure 1c) contained trapped air, which makes the Cassie–Baxter state^41^ exist. Unusual tribology of the surface of the film is generated due to the Cassie-like air-trapping wetting, providing easy sliding of water, which is important for the good performance of the T-TENG.

### Antireflection coating reinforced transmittance

**Figure 2:**
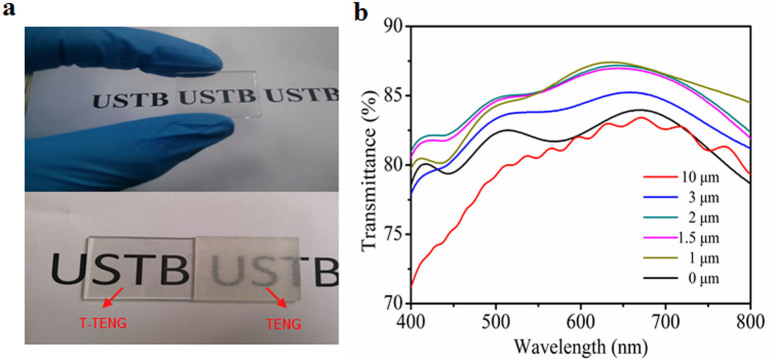
Characterization of transmittance of the T-TENG. (a) The photograph of a T-TENG placed above a paper with alphabets on it. The bottom is the comparison of the T-TENG and the TENG composed of a commercial 50 μm PTFE film. (b) The UV-vis spectra of the T-TENG with different thickness of the PTFE film.

Figure 2a displays the photograph of a T-TENG placed above a paper with alphabets on it and the bottom is the comparison of the T-TENG and the TENG composed of a commercial 50 μm PTFE film, from which we can find the T-TENG (left) is more transparent than the TENG (right), which indicates the high transparency of the T-TENG.

To systematically characterize the transparency of the fabricated devices with different thicknesses of the films, UV-vis spectroscopy was used to investigate the transmittances of our devices, as shown in Figure 2b. The maximum transmittance is 83.41% for the FTO glass substrate, 85.24%, 87.18%, 86.98% and 87.41% for the devices with different films thicknesses about 3, 2, 1.5, 1 μm, respectively. Compared with the FTO glass substrate, the increases of transmittances of the aforementioned devices can be explained by the role of the PTFE films acting as antireflection coatings, which are widely used to reduce the surface reflection of optical devices, thus increased the transmittances of the substrates. The interference of the reflected light from the substrate-coating and coating-air interfaces is the principle of the antireflection. So, the refractive index of the coatings between that of the substrate and the air is necessary. For an ideal homogeneous single-layer antireflection coating, two conditions should be fulfilled^42^:
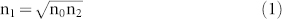

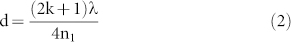
where n_0_, n_1_, n_2_ are the refractive indices of the air, PTFE film and FTO, which are equal to 1, 1.35, 1.8, respectively. λ is the wavelength of the incident light. K is equal to 0, 1, 2, 3 …. For the fabricated PTFE film, the aforementioned two conditions can be fulfilled and that is why the transmittances of the devices with PTFE films with thickness below 3 μm are larger than that of the glass substrate. In addition, the transmittance of the T-TENG with PTFE film thickness about 10 μm (Figure 2b) is a little smaller than that of the glass substrate, which can be explained by that the thickness is too large and more light is absorbed by the film.

### Electricity generation process

**Figure 3:**
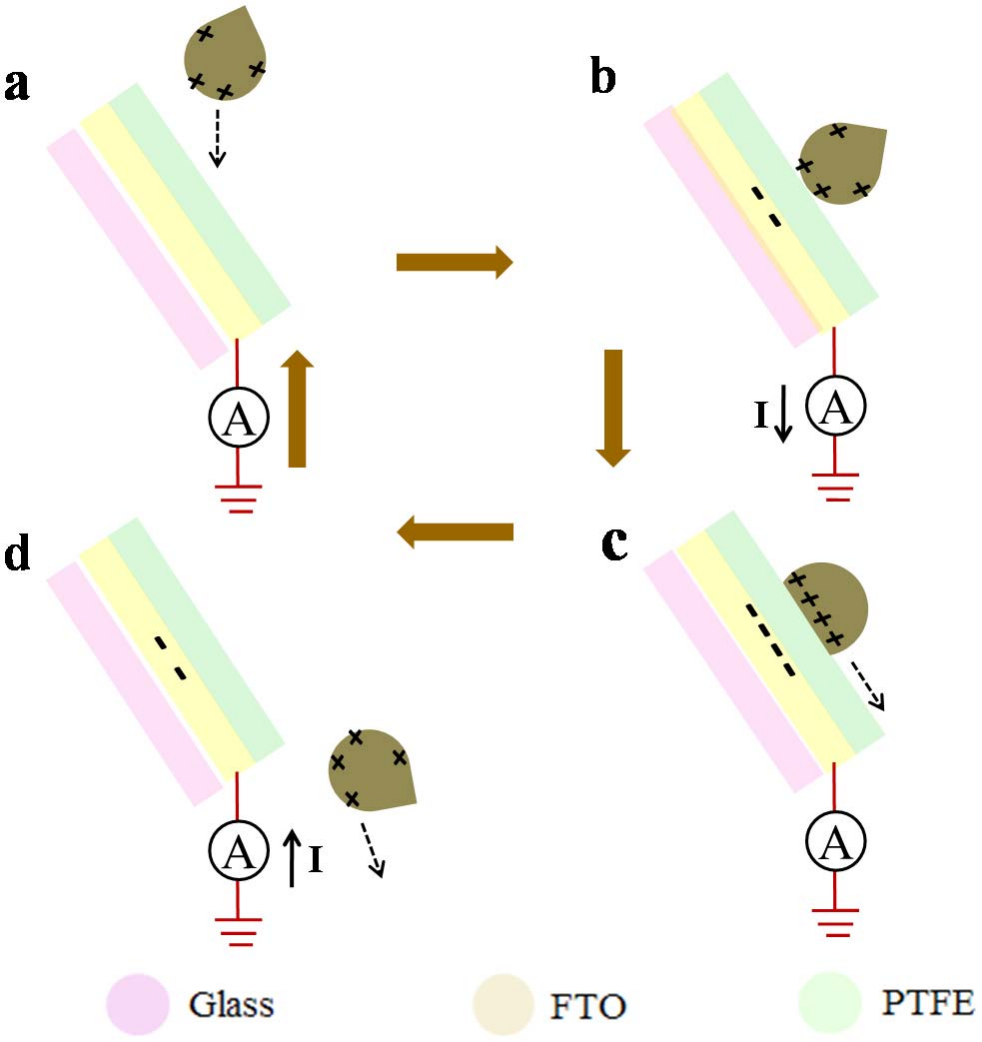
Working mechanism of the T-TENG. (a) Water drop falls toward the charged surface of the PTFE film. (b) Water drop contacts with the film. (c) Water drop slides down the film. (d) Water drop leaves the film surface.

The flowing tap water from a household faucet was used to drive the T-TENG in our experiment. The working principle of the T-TENG will be explained as single-electrode mode. When water drop fall from the sky or travel through an insulating tube, triboelectricity will be generated thus the water drop will be charged. For simplification, we choose positively charged water drop to illustrate the process of energy generation (Figure 3a). A positive electric potential difference will be created between the FTO electrode and ground, as the positively charged water drop approaches the PTFE film (Figure 3b). Electrons will be transferred from ground to FTO electrode to balance the potential difference and finally achieve equilibrium state (Figure 3c). As a result, an instantaneously positive current is produced. When the water drop leaves the film, a negative electric potential difference between the ground and the FTO electrode will be formed. Electrons will be transferred from the FTO electrode to the ground (Figure 3d), and another new equilibrium state is obtained. If the flowing water contact and leave the T-TENG periodically (Figure 3a–d), continuous outputs can be obtained.

### Electric measurement

**Figure 4:**
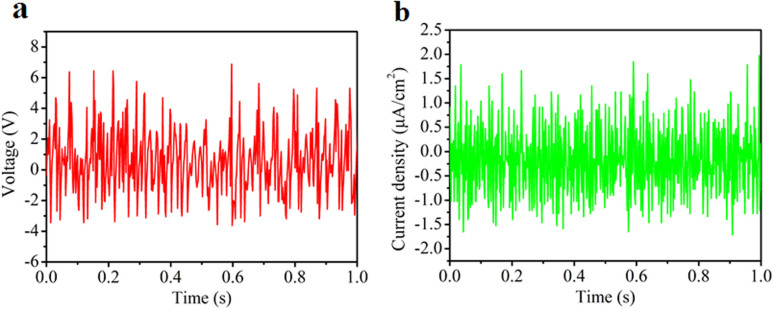
Results of electric measurements. (a) Output voltage and (b) output current density of the T-TENG generated from flowing water.

To demonstrate the potential applications of the T-TENG which can be used to harvest the water-related energy from the environment in our daily life, the flowing tap water from a household faucet was applied to drive the T-TENG. The flow rate of the water was set at around 93 ml/s and the distance between the T-TENG and the faucet was 25 cm. The output open-circuit voltage (V_oc_) and current density (J_sc_) were measured to evaluate the performance of the T-TENG. As shown in Figure 4, the peak-to-peak value of V_oc_ could reach 10 V and the maximum output current density of 2 μA/cm^2^ can be obtained. Besides, the influence of the flow rate on the outputs of the T-TENG was also investigated (see Supplementary Fig. S1). The output voltage increased with the water flow rate increasing from 0 ml/s to 84 ml/s, and then kept almost constant with the flowing rate adding up.

**Figure 5:**
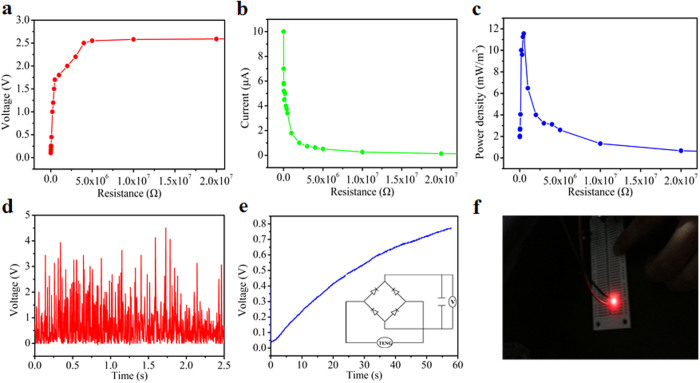
Dependence of the output of the T-TENG on external load and storage of the harvested energy from the T-TENG. (a) Output peak voltage, (b) output peak current and (c) output power density dependence on the resistance of external load. (d) Rectified voltage by a full-wave diode bridge. (e) The measured voltage of a 22 μF capacitor charged by the fabricated T-TENG. Inset: the equivalent circuit to store the harvested energy. (f) Digital photographs when the light emitting diode was lit up by capacitors.

For different applications, external loads with variable resistance will be connected with the energy harvester, thus the output would deviate from the open-circuit and short-circuit conditions. Considering this point, resistors were connected with the T-TENG to systematically study the reliance of the output performance on different external loads with a flowing rate of the water stream from the household faucet to be 93 ml/s. As demonstrated in Figure 5a, b, the output voltage increases from 0.1 V to 2.75 V when the load varies from 10 KΩ to 5 MΩ, while the output current follows a reversed tendency decreasing from 10 μA to 0.07 μA under the same external loads. Correspondingly, the instantaneously power density (P/A = U^2^/RA, A is the effective area of the T-TENG) reaches the maximum value of 11.56 mW/m^2^ at a load of 0.5 MΩ, as depicted in Figure 5c. The measurement results reveal that the fabricated T-TENG is efficient for harvesting energy from flowing water provided that the load has a resistance on the order of a fraction of megaohm.

### Charging capacitor

There is a problem we may face that the TENG give an alternating current output pulses, which cannot be used to power electric devices directly in most cases on account of that the devices usually need a constant bias voltage or current. The storage unit, such as a supercapacitor, battery or capacitor can store the pulse energy to supply a continuous power. Then an integrated full-wave rectifying bridge comprised of a rectifying bridge, a T-TENG and a capacitor of 22 μF which is shown in the inset of Figure 5e was applied to further transform the alternating current output to pulse output in the same directions. The obtained rectified output voltage of the T-TENG is shown in Figure 5d from which one can find the output has been converted to direct current signals. During the charging process, the voltage across a capacitor was monitored and we find that it takes less than 1 minute to charge the capacitor to 0.7 V with the T-TENG driven by water stream at a flow rate of 93 ml/s, as depicted in Figure 5e. The energy stored in the capacitors was then used to light up a light emitting diode for 1 s with three charged capacitors connected in series, as shown in Figure 5f, which indicates the feasibility of harvesting energy from flowing water by the T-TENG.

### Operation in ambient environment

**Figure 6:**
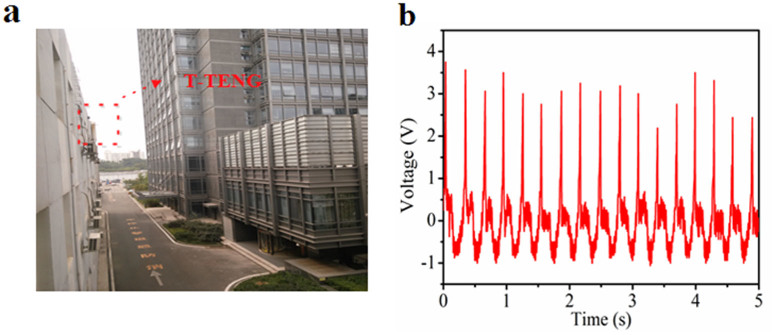
Demonstration of the potential application of the T-TENG for harvesting energy from raindrop. (a) The photograph of a T-TENG attached on the window glass. (b) Output voltage of the T-TENG on the window glass when impacted by the water-drops from the washing bottle. Photograph taken by Qijie Liang.

The high transparency of the T-TENG makes it possible that being applied on our building and vehicle for harvesting electrostatic energy from rain. We integrated our T-TENG with window glass. Figure 6a shows the photograph of T-TENG adhered on the surface of a window glass which also certified the high transparency of the T-TENG. When deionized water-drops from the washing bottle impacts the T-TENGs on the window glass circularly, the outputs are generated, which is shown in Figure 6b. A positive pulse output is achieved when the water-drop falls on the T-TENG, while a negative pulse output is obtained when the water-drop leaves the T-TENG, which confirms the working mechanism of the T-TENG for harvesting electrostatic energy of flowing water. The above results indicate that the T-TENG have the potential to be used in our daily life for harvesting energy from rain.

## Discussion

The T-TENG has distinct basic mechanism comparing with other technologies for power generation. It generates electricity on the basis of the combination of triboelectrification and charge-induction effect. Recently, several studies^32^,^33^ have shown that the triboelectric nanogenerator can be used to harvest water-related energy. In view of the practical application environment of the triboelectric nanogenerator for harvesting water-related energy, the transparent characteristic of the designed device will be necessary in many cases, for example, integrating with solar-cell, building glass, vehicle glass and so on. So we designed a T-TENG which composed of a transparent conductive FTO electrode and a transparent PTFE film to meet the demand. The thickness of commercial PTFE film is about tens of micrometers and the semi-transparent film is not appropriate for preparing highly transparent triboelectric nanogenerator. Therefore, we fabricated the PTFE films with different thicknesses by using diluted PTFE suspension. The smallest thickness of the as-prepared PTFE film is less than 1 μm, which contributes to the high transparency of the T-TENG.

The high transmittance of the T-TENG is not only caused by the thinness of the film, but also caused by the acting as an antireflection coating of the film. The introduction of antireflection coating to the T-TENG increases the transmittance of this kind of devices which can remind us of considering the using of the mechanism of antireflection coating to increase the transmittance of the T-TENG or other transparent devices.

The silicon-based solar cell is widely used in our living environment to transform the sunlight to electricity, but it is limited by daytime and climate. In the future, the developed T-TENG can be integrated with the silicon-based solar cell to harvest water-related energy from rain which is ignored by us and the high transmittance of the T-TENG will make this possible. Besides, the T-TENG can also be applied to our building and vehicle for harvesting water-related energy to power other electronics which would be beneficial for the construction of smart home system and smart car system.

To increase the capacity of the T-TENG for harvesting water-related energy, the future efforts will be focused on: (a) the preparation of nanostructures on the film surface which can increase the effective contact area between water and the film^43^. (b) the integration of large-scale devices which can multiply the output of the T-TENG.

There are several advantages for this designed triboelectric nanogenerator. i) It is a transparent triboelectric nanogenerator, which is important for practical applications in our daily life, for example, the T-TENG can be applied to our window glass for harvesting water-related energy. ii) The thickness of the fabricated PTFE film is decreased to 1 μm, which is of great significance for the high transparency of the fabricated devices. iii) Because of the PTFE film served as an antireflection coating, the as-prepared devices are more transparent than the individual glass substrate. iv) The fabrication process is simple and low-cost, which exhibits great advantages in industrial production and practical applications.

In summary, a highly transparent triboelectric nanogenerator (T-TENG) have been developed and demonstrated to harvest the electrostatic energy of flowing water. Upon the impact of the water from a common household faucet, the output peak-to-peak V_oc_ and output J_sc_ achieved 10 V and 2 μA/cm^2^, respectively. The instantaneous output power density from the T-TENG reached 11.56 mW/m^2^ when connected to a load resistor of 0.5 MΩ. Commercial capacitors were charged and light emitting diode was illuminated by the rectified output of the T-TENG. In addition, we introduced the mechanism of antireflection coating to increase the transmittance of the T-TENG, which can provide guidance for future design of similar transparent devices. With the PTFE film playing a role of antireflection coating, the transmittance of the fabricated device covered by PTFE film with thickness of 1 μm is 87.41%, larger than that of glass substrate of 83.41%. All these features show that the T-TENG have the potentials to integrate with silicon-based solar cell, building glass and car glass for harvesting the energy from water in our environment which is beneficial for the construction of smart home system and smart car system.

## Methods

### Fabrication of a T-TENG

Before the preparation of the PTFE film, the purchased FTO glass substrate cut into a rectangular (3 cm * 2.5 cm) with a thickness of 2.2 mm was ultrasonically cleaned in acetone, ethanol and isopropyl alcohol for 10 minutes, respectively. After blown dry with nitrogen, the substrate was partially covered with adhesive tape for acting as an electrode. Then, the FTO glass substrates were spin-coated with a commercial non-purified PTFE suspension diluted proportionally at 1000 rpm for 10 s to form the PTFE films with different thicknesses. After a conventional vacuum process for removing the remained air, the film-covered substrate was heated in an oven at 60°C for 30 minutes. Subsequently, the precursor film was annealed at 380°C for 10 minutes. Finally, the conducting wire was connected to the electrode for subsequent measurements. The effective dimension of the T-TENG is 2 cm * 2.5 cm.

### Characterization

Field emission scanning electron microscopy (FEI Quanta 3D) was used to measure the morphology and thickness of the PTFE film. The surface morphology of the PTFE film was characterized by the Atomic Force Microscopy (Multi-mode 3, Bruker). The UV-vis spectroscopy method with Agilent Cary 5000 spectrophotometer was used to characterize the transmittances of the fabricated devices and the reference was set to be atmosphere. During the test, the flowing water was applied to the T-TENG for the measurement of typical electrical output. A digital oscilloscope (DS4052, RIGOL) was used to test the electric outputs of the T-TENG. The entire test was carried out in ambient environment.

## Author Contributions

Q.L., X.Y., Y.G. and Y.Z. designed the T-TENG. Q.L. and K.Z. fabricated the T-TENG. Q.L., M.L. and S.L. analyzed the experimental data. Q.L., X.Y. and Y.Z. prepared the manuscript. X.Z. participated to the test of contact angle. All authors contributed to reviewing the manuscript.

## Supplementary Material

Supplementary Information Supplementary Information
