# Supplementary material for: Highly transparent triboelectric nanogenerator for harvesting water-related energy reinforced by antireflection coating
Source: Sci Rep. 2015 Mar 13;5:9080. doi: 10.1038/srep09080 (PMC4357854; doi:10.1038/srep09080)
Supplement: Supplementary Information [file srep09080-s1.pdf]

Highly transparent triboelectric nanogenerator for harvesting water-related energy reinforced by antireflection coating

Qijie Liang[1], Xiaoqin Yan[1,*], Yousong Gu[1], Kui Zhang[1], Mengyuan Liang[1], Shengnan Lu[1], Xin Zheng[1], Yue Zhang[1,2,*]

[1]State Key Laboratory for Advanced Metals and Materials, School of Materials Science and Engineering, University of science and technology Beijing, Beijing, 100083, China

[2] Key Laboratory of New Energy Materials and Technologies, University of Science and Technology Beijing, Beijing, 100083, China

* Corresponding author: Key Laboratory of New Energy Materials and Technologies, University of science and technology Beijing, Beijing, 100083, China.

E-mail: yuezhang@ustb.edu.cn (Y. Zhang), xqyan@mater.ustb.edu.cn (X. Yan).

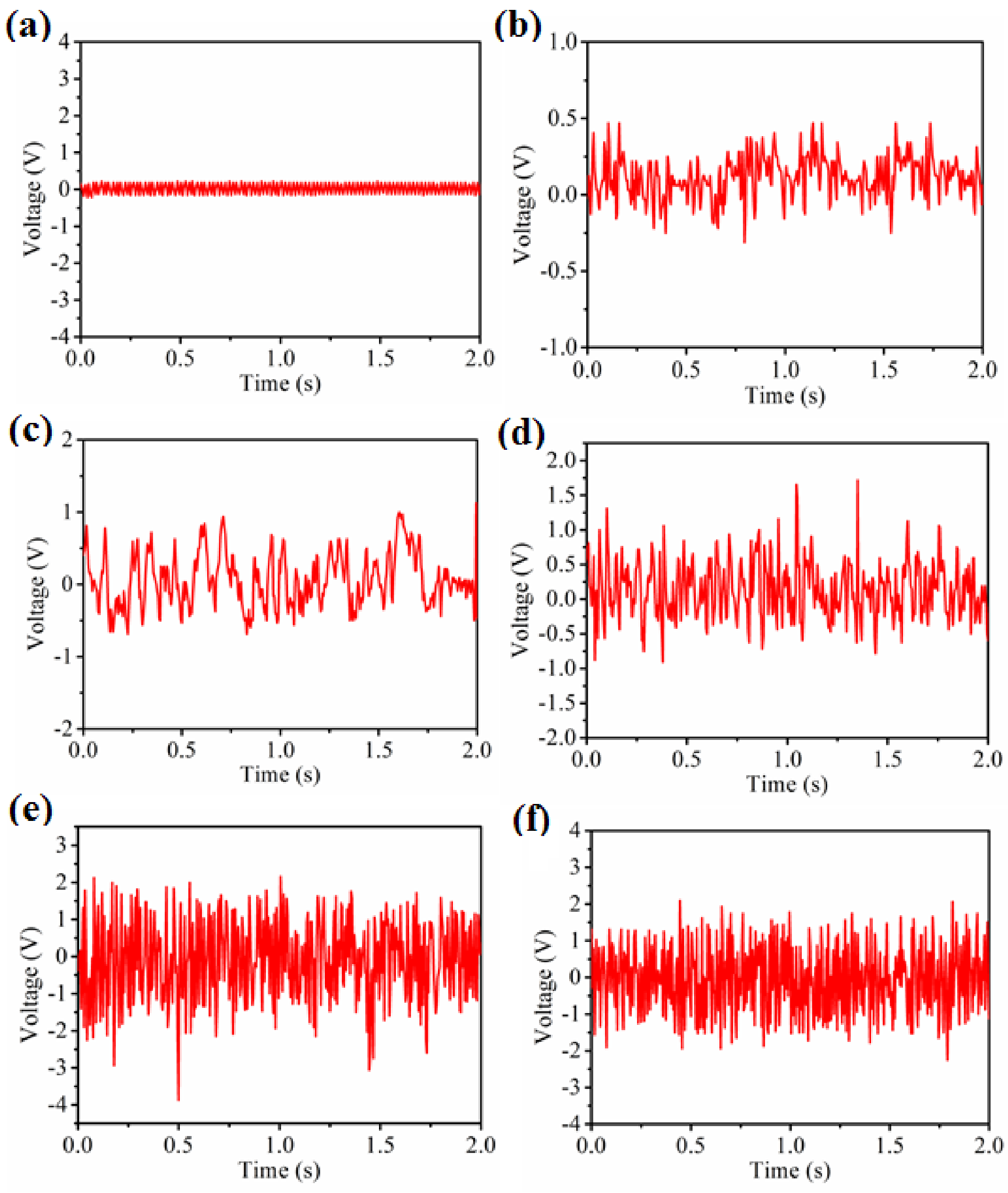

Figure S1 | **Influence of the flow rate on the output of the T-TENG.** **(a)** – **(f)** Output voltage of the T-TENG with flow rate of 0, 14, 35, 65, 84, 110 ml/s, respectively.
